# Epidemiological investigation and intergenerational clinical characteristics of 24 coronavirus disease patients associated with a supermarket cluster: a retrospective study

**DOI:** 10.1186/s12889-021-10713-z

**Published:** 2021-04-01

**Authors:** Suochen Tian, Min Wu, Zhenqin Chang, Yunxia Wang, Guijie Zhou, Wenming Zhang, Junmin Xing, Hui Tian, Xihong Zhang, Xiuli Zou, Lina Zhang, Mingxin Liu, Juan Chen, Jian Han, Kang Ning, Shuangfeng Chen, Tiejun Wu

**Affiliations:** 1 Intensive Care Unit, Liaocheng People’s Hospital, Liaocheng, 252000 Shandong China; 2 Department of Healthcare Associated Infection, Liaocheng People’s Hospital, Liaocheng, 252000 Shandong China; 3 Department of Hemodialysis, Liaocheng People’s Hospital, Liaocheng, 252000 Shandong China; 4 Intensive Care Unit, Liaocheng Infectious Diseases Hospital, Liaocheng, 252000 Shandong China; 5 Intelligence Library Center, Liaocheng People’s Hospital, Liaocheng, 252000 Shandong China; 6 Department of Respiratory Medicine, Liaocheng People’s Hospital, Liaocheng, 252000 Shandong China; 7 Department of Pulmonology, The Affiliated Hospital of Shandong University of TCM, Jinan, 250000 Shandong China; 8 Department of Respiratory Medicine, The First Affiliated Hospital of Shandong First Medical University, Jinan, 250000 Shandong China; 9 Department of Central Laboratory, Liaocheng People’s Hospital, Liaocheng, 252000 Shandong China

**Keywords:** COVID-19, Cluster, Epidemiology, Generation, Clinical characteristics

## Abstract

**Background:**

In view of the ongoing coronavirus disease (COVID-19) pandemic, it remains unclear whether the severity of illness and time interval from symptom onset to release from quarantine differ between cases that originated from clusters and cases reported in other areas. This study aimed to assess epidemiological and intergenerational clinical characteristics of COVID-19 patients associated with cluster outbreaks to provide valuable data for the prevention and control of COVID-19.

**Methods:**

We identified the first employee with COVID-19 at a supermarket and screened the close contacts of this index patient. Confirmed cases were divided into two groups according to the generation (first generation comprising supermarket employees [group A] and second or third generations comprising family members or friends of the supermarket employees [group B]). The epidemiological and clinical characteristics of the two groups were retrospectively compared.

**Results:**

A total of 8437 people were screened, and 24 COVID-19 patients were identified. Seven patients (29.2%) were asymptomatic; three patients were responsible for six symptomatic cases. The interval from the confirmation of the first case to symptom onset in symptomatic patients was 5–11 days. The clinical manifestations of symptomatic patients upon admission were non-specific. All patients (including the seven asymptomatic patients) were admitted based on chest computed tomography features indicative of pneumonia. There were 11 cases in group A (first generation) and 13 cases in group B (second generation, 11 cases; third generation, 2 cases), with no significant differences in clinical and epidemiological characteristics between the two groups, except for sex, duration from symptom onset to hospitalization, and underlying disease (*P* > 0.05).

**Conclusions:**

For cluster outbreaks, it is important to comprehensively screen close the contacts of the index patient. Special attention should be paid to asymptomatic cases. The clinical management of cluster patients is similar to that of other COVID-19 patients.

## Background

Coronavirus disease (COVID-19) has spread globally. Several case series on clinical characteristics [1–4] and research articles on epidemiological characteristics of COVID-19 have been published [5–9].

Liaocheng city, located in the middle eastern region of China, far from the Hubei province and Wuhan city, had a record of 38 patients with confirmed COVID-19 during the current outbreak. Among those patients, 63.2% (*n* = 24) were associated with a supermarket cluster, which was the primary source of COVID-19 in non-outbreak areas. Compared with COVID-19 cases reported in other areas, it is unclear whether the pattern of clinical characteristics, such as the severity of illness and time interval from symptom onset to release from quarantine, in COVID-19 cases associated with cluster outbreaks are different. We aimed to characterize clinical and epidemiological features of COVID-19 cases associated with a cluster outbreak and determine whether there were intergenerational clinical variations in clinical symptoms.

## Methods

The identified patients were diagnosed and treated following the protocol issued by the National Health Commission of the People’s Republic of China and the National Administration of Traditional Chinese Medicine. According to the protocol, patients were divided into mild, moderate, severe, and critical groups depending on disease severity. The difference between patients with mild and those with moderate disease was the presence of pneumonia. Patients with respiratory dysfunction or multiple organ dysfunction syndrome were considered to have severe disease or be critically ill [10–12].

On January 22, 2020, a supermarket employee presented with a fever and underwent RT-PCR testing for severe acute respiratory syndrome coronavirus 2 (SARS-CoV-2) twice at the fever clinic of a designated hospital. The results were all positive, and COVID-19 was diagnosed. The patient was admitted to a designated hospital on January 23, 2020, and the supermarket where he worked was shut down immediately. After the first confirmed case, all the close contacts of the patient were screened, including the patient’s cohabiting family members or friends who had visited the patient in the past week, followed by all supermarket employees and people who had visited the supermarket from January 15 to January 22, 2020. RT-PCR assays of nasal and pharyngeal swab specimens were routinely performed on screened subjects to determine whether they were asymptomatic cases. If a patient was diagnosed with COVID-19, all persons who were in close contact with the patient over the past week, especially the family and close friends, were screened. All patients with confirmed COVID-19 underwent chest computed tomography (CT), centralized monitoring, and treatment at designated hospitals.

The incubation period of these patients was difficult to determine because they had no history of sojourn in the epidemic area. It was also challenging to confirm with certainty the source of SARS-CoV-2 infection, which was assumed to be the supermarket employees. Interestingly, no supermarket customers were diagnosed with COVID-19 during this screening, but the confirmed infection cases were found among the family members or friends of the confirmed infected supermarket employees. Therefore, the supermarket employees were identified as first-generation cases and their family members and friends as second-generation cases. Confirmed cases resulting from close contact with the confirmed family members or friends were regarded as third-generation cases.

The criteria to release patients from quarantine depended on the improvement of clinical manifestations, resolution of acute exudative lesions, and two consecutive negative SARS-CoV-2 RT-PCR tests from respiratory tract specimens (sample collection at least 24 h apart) [10–12].

This study analyzed the clinical characteristics between 24 COVID-19 patients associated with a supermarket cluster outbreak and COVID-19 patients reported in other areas. For this purpose, patients were divided into two groups according to the generation (first generation comprising supermarket employees [group A], and second and third generations comprising family members and friends of supermarket employees [group B]). The clinical characteristics of the two groups were compared. All data and images in this study were sourced from the information system of the Liaocheng Infectious Diseases Hospital.

Data were analyzed using SPSS version 23.0 (IBM Corp., Armonk, NY, USA). Differences were considered significant if the *P*-value was < 0.05. Continuous variables are shown as mean ± standard deviation or medians and interquartile ranges. Categorical variables are presented as counts and percentages for each group. We used a *t*-test or Wilcoxon rank-sum test to analyze continuous variables. The chi-square test or Fisher’s exact test was used to analyze categorical variables.

## Results

During the outbreak, 8437 people (93 relatives or friends of confirmed cases, 120 supermarket employees, and 8224 supermarket customers) were screened. Among 24 identified cases of COVID-19, 11 were supermarket employees, and 13 were family members and friends of the 11 employees (ten family members and three friends). No supermarket customers tested positive for SARS-CoV-2. The total infection rate in the screened population was 0.3%. The infection rate of the patients’ family members’ and friends’ was 14.0%, and that of supermarket employees was 9.2%.

Seven patients were asymptomatic. Six patients were supermarket employees, of whom three spread the disease to six family members or friends who then became symptomatic.

**Fig. 1:**
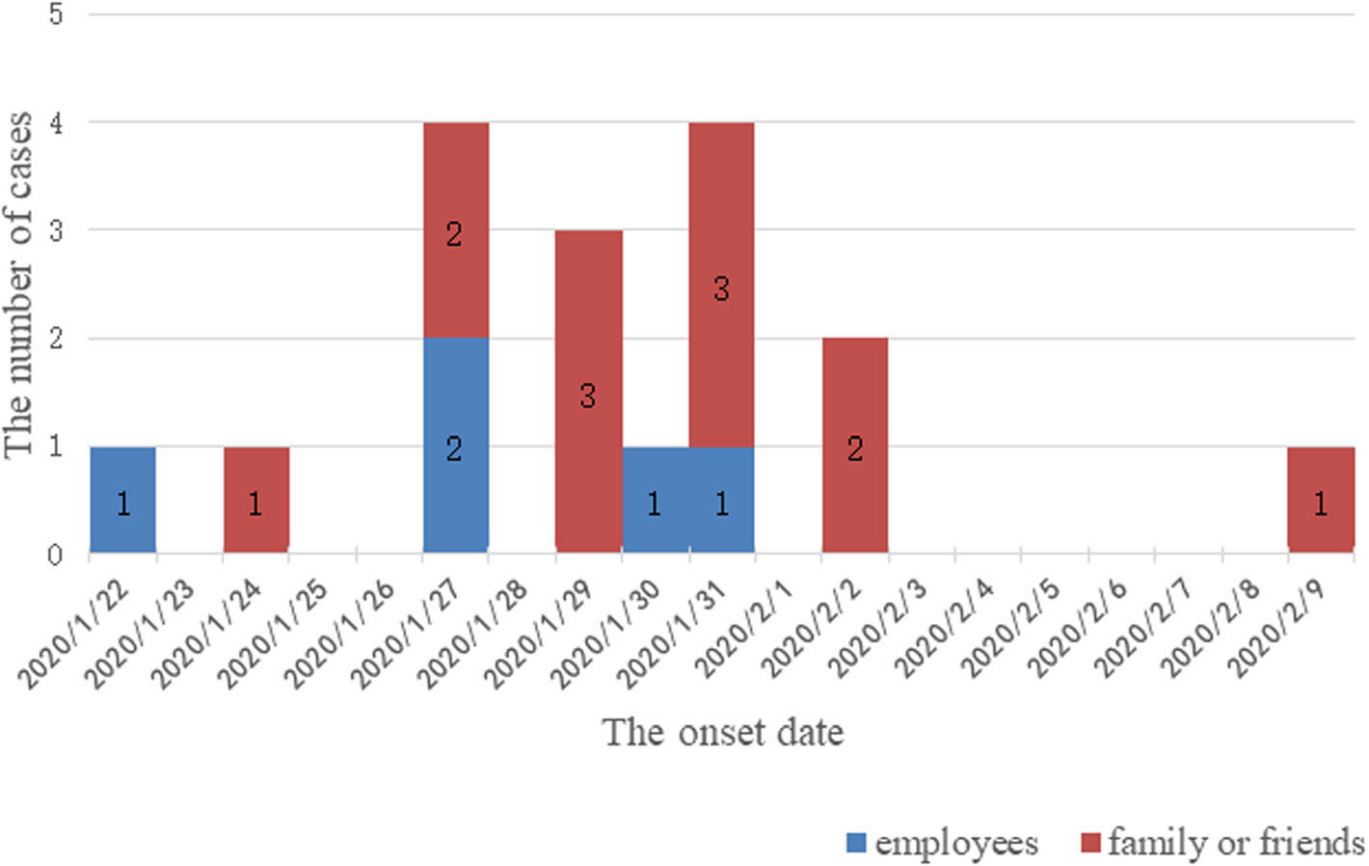
Time distribution of confirmed symptomatic coronavirus disease (COVID-19) patients associated with a supermarket cluster

**Fig. 2:**
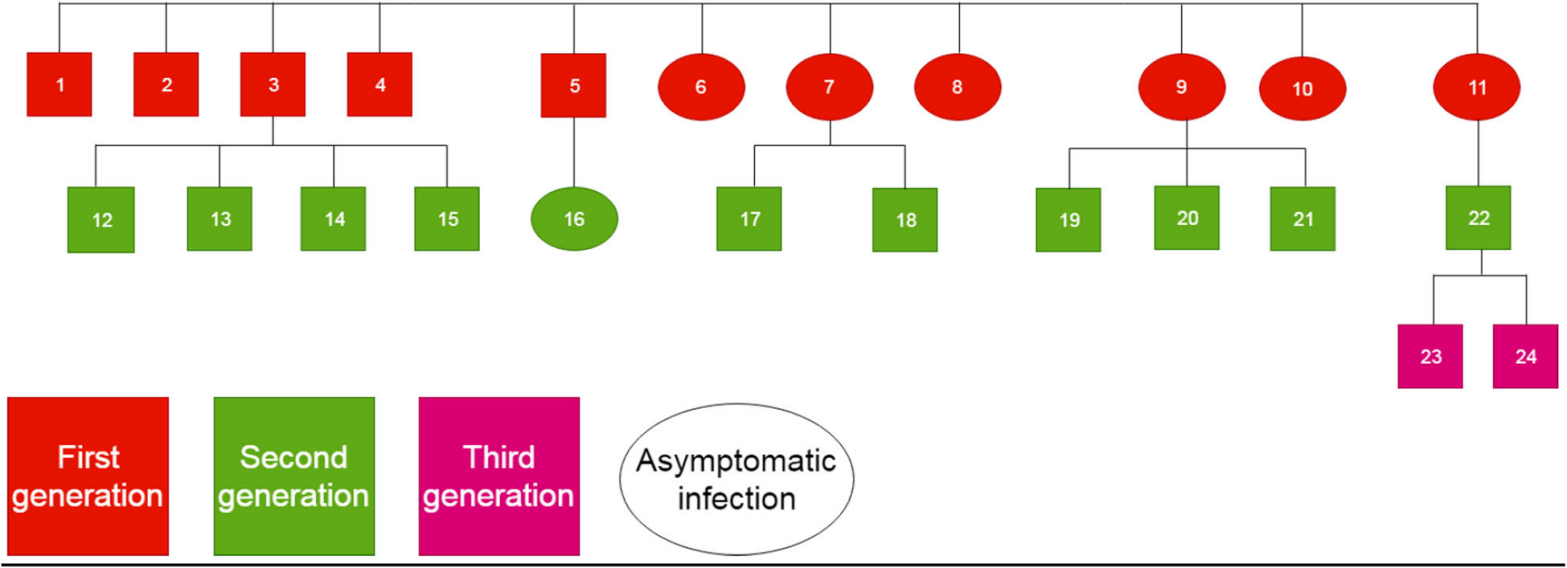
Transmission chain and intergenerational relationship

The interval from the confirmation of the first case to symptom onset in symptomatic patients was 5–11 days (Fig. 1). Three generations of cases were generated in the clusters, including 11 cases in the first generation (45.8%), 11 in the second generation (45.8%), and two in the third generation (8.3%) (Fig. 2).

The first confirmed patient was diagnosed with COVID-19 after the onset of symptoms. The period between symptom onset and release from quarantine was the longest in this patient (34 days). The shortest period was 8 days and corresponded to a patient was diagnosed during contact tracing (case 11). In case 3, the patient experienced fever, and the patient’s parent, spouse, and two children were all diagnosed with COVID-19 after all three experienced fever on the same day. However, the time from symptom onset to release from quarantine was different among the five people, ranging from 17 to 30 days. In case 9, the patient was asymptomatic and was confirmed with COVID-19 during screening but had infected three family members who were symptomatic.

**Fig. 3:**
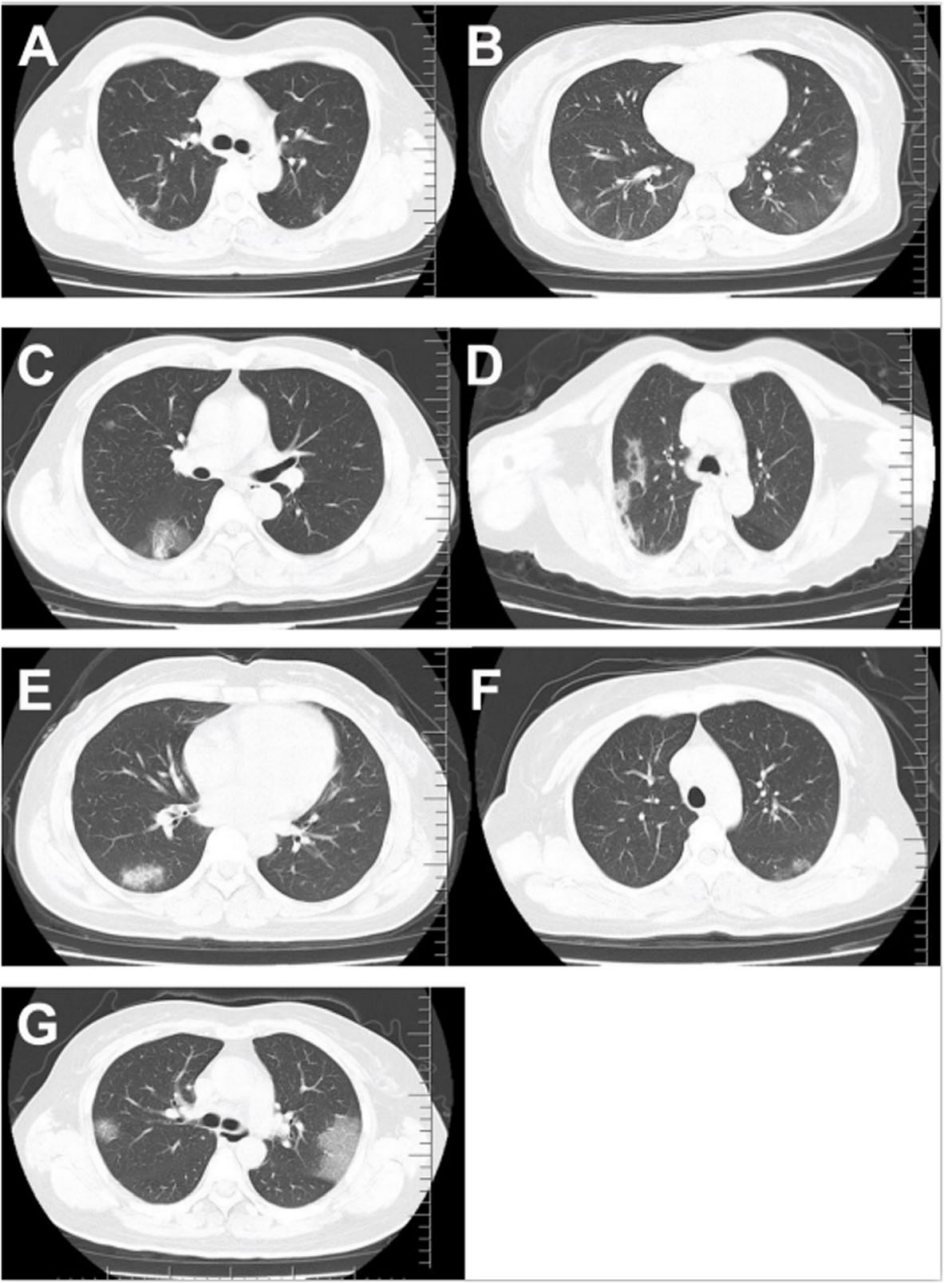
Computed tomography findings of 7 asymptomatic coronavirus disease (COVID-19) patients on admission

Twenty-three of the 24 patients in this group had moderate disease. However, according to the Pneumonia Severity Index (PSI), 83.3% of the patients had mild grade I and II disease, four patients had grade III and IV disease but did not develop severe pneumonia or critical illness. One elderly patient with grade II disease had a history of diabetes and developed severe disease due to acute respiratory distress syndrome 20 days after the diagnosis of COVID-19 [13]. Regarding the symptoms reported upon admission (in addition to asymptomatic patients), the most common one was a cough, followed by fever. Other symptoms were successive shortness of breath, sore throat, gastrointestinal symptoms, and fatigue. Regarding hematological tests performed upon admission, besides white blood cell and lymphocyte counts, the tests showed results that were normal or within the reference range. It was noteworthy that all patients (including seven asymptomatic patients, Fig. 3) were admitted based on chest CT features consistent with pneumonia, often with bilateral infiltration. Regarding treatment, all patients received 2–3 antiviral drugs, and some additionally received antibiotics (17 patients), hormones (five patients), and immunomodulators (15 patients) during hospitalization. In addition to one patient with severe COVID-19 treated with high-flow nasal cannula oxygen therapy, all other patients received oxygen through a nasal catheter (normal flow). All patients received traditional Chinese medicine (TCM), including Chinese medicine preparations, acupuncture, and moxibustion (Tables 1 and 2).

**Table 1:** Clinical characteristics of the study patients on admission

Characteristics	All patients (*n* = 24)	Group		
		A (*n* = 11)	B (*n* = 13)	*P*-value
Age (y)	48 ± 1.73	42.05 ± 9.46	53 ± 2.09	0.11
Gender				
Male (%)	9/24 (37.50)	1/11 (9.09)	8/13 (61.54)	0.013
Female (%)	15/24 (62.50)	10/11 (90.91)	5/13 (38.46)	
Duration from onset to hospitalization (d)	5.5 (7)	3 (5)	6 (4)	0.011
PSI	0 (57.25)	0 (44)	45 (72.5)	0.252
I (%)	16/24 (66.67)	9/11 (81.82)	7/13 (53.85)	
II ≤70 (%)	4/24 (16.67)	1/11 (9.09)	3/13 (23.08)	
III 71–90 (%)	3/24 (12.50)	1/11 (9.09)	2/13 (15.38)	
IV 91–131 (%)	1/24 (4.17)	0/11 (0)	1/13 (7.69)	
V > 130 (%)	0/37 (0)	0/19 (0)	0/18 (0)	
Underlying disease				
Any (%)	6/24 (25.00)	0/11 (0)	6/13 (46.15)	0.016
Hypertension (%)	2/24 (8.33)	0/11 (0)	2/13 (15.38)	0.482
Coronary heart disease (%)	2/24 (8.33)	0/11 (0)	2/13 (15.38)	0.482
Diabetes (%)	1/24 (4.17)	0/11 (0)	1/13 (7.69)	1
Cirrhosis, liver cancer (%)	1/24 (4.17)	0/11 (0)	1/13 (7.69)	1
Symptoms				
Asymptomatic (%)	7/24 (29.17)	6/11 (54.55)	1/13 (7.69)	0.023
Fever (%)	15/24 (62.50)	5/11 (45.45)	10/13 (76.92)	0.206
Fatigue (%)	5/24 (20.83)	1/11 (9.09)	4/13 (30.77)	0.327
Headache (%)	1/24 (4.17)	1/11 (9.09)	0/13 (0)	0.458
Nasal congestion (%)	2/24 (8.33)	0/11 (0)	2/13 (15.38)	0.482
Sore throat (%)	6/24 (25.00)	2/11 (18.18)	4/13 (30.77)	0.649
Cough (%)	17/24 (70.83)	8/11 (72.73)	9/13 (69.23)	1
Hemoptysis (%)	1/24 (4.17)	0/11 (0)	1/13 (7.69)	1
Shortness of breath (%)	10/24 (41.67)	3/11 (27.27)	7/13 (53.85)	0.240
Vomiting or diarrhea (%)	6/24 (25.00)	3/11 (27.27)	3/13 (23.08)	1
Pain in a muscle or joint (%)	1/24 (4.17)	0/11 (0)	1/13 (7.69)	1
Laboratory findings				
WBC < 4.0 × 10^9^/L (%)	7/24 (29.17)	3/11 (27.27)	4/13 (30.77)	1
L < 1.1 × 10^9^/L (%)	14/24 (58.33)	6/11 (54.55)	8/13 (61.54)	1
L ≤ 0.6 × 10^9^/L (%)	7/14 (50.00)	3/6 (50.0)	4/8 (50.00)	1
SAA > 10 mg/L (%)	15/24 (62.50)	5/11 (45.45)	10/13 (76.92)	0.206
CRP > 5 mg/L (%)	12/24 (50.00)	3/11 (27.27)	9/13 (69.23)	0.1
PCT > 0.5 ng/ml (%)	2/24 (8.33)	2/11 (18.18)	0/13 (0)	0.199
LDH > 250 U/L (%)	5/24 (20.83)	1/11 (9.09)	4/13 (30.77)	0.327
Alb < 40 g/L (%)	17/24 (70.83)	8/11 (72.73)	9/13 (69.23)	1
AST > 40 U/L (%)	2/24 (8.33)	1/11 (9.09)	1/13 (7.69)	1
ALT > 40 U/L (%)	1/24 (4.17)	1/11 (9.09)	0/13 (0)	0.458
TBL > 17.1 mmol/L (%)	9/24 (37.50)	3/11 (27.27)	6/13 (46.15)	0.423
D-dimer > 0.5 mg/L (%)	6/24 (25.00)	3/11 (27.27)	3/13 (23.08)	1
Fib > 4 g/L (%)	6/24 (25.00)	3/11 (27.27)	3/13 (23.08)	1
ESR ≥20 mm/h (%)	18/24 (75.00)	8/11 (72.73)	10/13 (76.92)	1
Chest CT findings				
Pneumonia (%)	24/24 (100)	11/11 (100)	13/13 (100)	–
Bilateral infiltration (%)	18/24 (75.00)	7/11 (63.64)	11/13 (84.62)	0.357
Unilateral infiltration (%)	6/24 (25.00)	4/11 (36.36)	2/13 (15.38)

**Table 2:** Disease severity classification among patients and complications and treatment measures before release from quarantine

Characteristics	All patients (*n* = 24)	Group		
		A (*n* = 11)	B (*n* = 13)	*P*-value
Disease severity				0.776
Mild (%)	0/24 (0)	0/11 (0)	0/13 (0)	
Moderate (%)	23/24 (95.83)	11/11 (100)	12/13 (92.31)	
Severe (%)	1/24 (4.17)	0/11 (0)	1/13 (7.69)	
Critical (%)	0/24 (0)	0/11 (0)	0/13 (0)	
Complications				
ARDS (%)	1/24 (4.17)	0/11 (0)	1/13 (7.69)	1
Treatment				
Antibiotics	17/24 (70.83)	9/11 (81.82)	8/13 (61.54)	0.386
Antifungal drugs	1/24 (4.17)	0/11 (0)	1/13 (7.69)	1
Antiviral drugs (%)	24/24 (100)	11/11 (100)	13/13 (100)	–
Glucocorticoids (%)	5/24 (20.83)	2/11 (18.18)	3/13 (23.08)	1
Albumin (%)	11/24 (45.83)	2/11 (18.18)	9/13 (69.23)	0.019
Immunoglobulin (%)	6/24 (25.00)	2/11 (18.18)	4/13 (30.77)	0.649
Thymosin (%)	15/24 (62.50)	7/11 (63.64)	8/13 (61.54)	1
Oxygen therapy (%)	11/24 (45.83)	4/11 (36.36)	7/13 (53.85)	0.444
Common (%)	10/11 (90.91)	4/4 (100)	6/7 (85.71)	1
HFNC (%)	1/11 (9.09)	0/4 (0)	1/7 (14.29)	
TCM (%)	24/24 (100)	11/11 (100)	13/13 (100)	–

There were more men in group B than in group A. Furthermore, the time from symptom onset to hospital admission was longer, and there were more underlying diseases and more patients treated with albumin (*P* < 0.05) in group B than in group A. However, there were no significant differences in other indicators (Tables 1, 2, 3).

**Table 3:** Clinical outcomes of patients

Outcomes	All patients (*n* = 24)	Group		
		A (*n* = 11)	B (*n* = 13)	*P*-value
Hospitalization duration (d)	16 ± 6.15	16 ± 6.66	16 ± 5.96	1
Duration from onset to release from quarantine (d)	21.04 ± 6.77	19.09 ± 7.92	22.69 ± 5.41	0.201
Blood findings at the time of release from quarantine				
WBC < 4.0 × 10^9^/L (%)	3/24 (12.50)	1/11 (9.09)	2/13 (15.38)	1
L < 1.1 × 10^9^/L (%)	14/24 (58.33)	7/11 (63.64)	7/13 (53.85)	0.697
L ≤ 0.6 × 10^9^/L (%)	4/14 (28.57)	1/7 (14.29)	3/7 (42.86)	0.559
SAA > 10 mg/L (%)	4/24 (16.67)	1/11 (9.09)	3/13 (23.08)	0.596

## Discussion

The control of COVID-19 associated with a cluster outbreak is key in the prevention and control of a larger outbreak [14, 15]. After the first case of this event was identified, all possible close contacts over the previous week were screened, and the supermarket was immediately shut down. For other confirmed patients, people who had been in close contact with that patient in the past week were immediately screened. These measures aided in the timely detection and control of the source of infection. This was the most important measure for effectively controlling the further spread of this outbreak.

The time of symptom onset in symptomatic patients was relatively uniform, and it was not clear whether this was related to the incubation period of COVID-19. There were three intergenerational relationship levels among all cases; however, transmission occurred only among two generations. Other studies on cluster outbreaks of COVID-19 were mostly caused by family clusters, and the number of cases was also small [9, 16, 17]. Therefore, further analysis of larger clusters was needed.

Asymptomatic cases have been attracting increased attention [16, 18, 19], not only because these cases are common but also because of their importance in spreading the infection. The current study showed similar results to those of previous studies. Asymptomatic cases were also common and caused infections in other individuals, such as in case 9.

Several patients had slightly higher PSI scores; this was due to the presence of an underlying disease and age. COVID-19 patients in this cluster did not meet the diagnostic criteria for severe disease or critical illness. A patient with a PSI score of grade II developed severe disease during hospitalization 20 days after the diagnosis of COVID-19 but was not directly related to SARS-CoV-2. Therefore, these COVID-19 patients associated with the supermarket cluster all had moderate disease. There was no significant difference in disease severity, and it was unclear whether COVID-19 patients associated with the cluster outbreak presented milder disease.

Changes in clinical manifestations after viral infection may imply mutations in the virus, especially after multiple replications. In general, the basic clinical characteristics of patients in this study group were similar to those of other COVID-19 patients [1, 2]. There were some differences in basic clinical characteristics, such as sex, time from symptom onset to hospitalization, and underlying disease, between the first generation of patients and the second and third. These differences might have been related to the timeliness of screening and the characteristics of the population.

It is worth noting that regardless of the presence of clinical symptoms, all the patients in this group had chest CT features suggestive of pneumonia, the most common of which was bilateral infiltration. This feature is different from the features of other viral respiratory infections [20, 21].

Regarding a treatment regimen, although no specific antiviral drugs have been proven effective, several studies have suggested the benefits of the early use of antiviral agents [22, 23]. The patients in this group received at least two antiviral drugs. All patients were treated routinely with TCM, and multiple studies have demonstrated its role in inhibiting coronavirus [24–26].

The most relevant indicator for the release of patients from quarantine in the group was two successive negative RT-PCR results for SARS-CoV-2. Therefore, the duration from symptom onset to release from quarantine reflected that the patient had shed the virus from its respiratory tract. There were intergenerational differences in the period from symptom onset to hospital admission and the underlying disease. All patients in the two groups were ultimately released from quarantine, and there was no significant difference in the duration from symptom onset to release from quarantine. This may have been due to the small number of patients in this group and the fact that they had mild/moderate disease. In addition, the first supermarket employee diagnosed with symptoms (case 1) presented the most extended period from symptom onset to release from quarantine. Case 3 was symptomatic and caused familial clustering of COVID-19, with the same symptoms but with a significantly different time until released from quarantine. This study emphasizes that factors influencing the duration of SARS-CoV-2-positivity are complex [27, 28] and require further research.

This study was limited by a few factors. First, the patients included in this analysis were associated with a cluster outbreak. A cluster epidemic is a particular form of infectious disease and may not mirror the natural spread. The number of patients was limited, and there were regional limitations. Therefore, these cases did not represent the characteristics of patients with sporadic COVID-19 in a wider area.

## Conclusions

From this study, we draw the following conclusions: 1) the clinical characteristics of COVID-19 patients associated with the supermarket cluster were similar to those of other COVID-19 patients; 2) disease severity was similar and there was intergenerational spread; 3) there was no significant difference in clinical characteristics among generations, and 4) sufficient attention should be paid to asymptomatic COVID-19 patients not only because of their higher proportion but also because of their potential to spread the disease. Additional intergenerational characteristics of COVID-19 associated with a cluster outbreak still require further research that includes more cases.

## Data Availability

With the permission of the corresponding author, we can provide participant data and material for further statistical analysis.

## Abbreviations

COVID-19: Coronavirus Disease
RT-PCR: Reverse Transcription Polymerase Chain Reaction
CT: Computed Tomography
SARS-CoV-2: Severe Acute Respiratory Syndrome Coronavirus 2
IQRs: Interquartile Ranges
PSI: Pneumonia Severity Index
TCM: Traditional Chinese Medicine
WBC: White Blood Cell
L: Lymphocyte
SAA: Serum Amyloid A
CRP: C-Reactive Protein
PCT: Procalcitonin
LDH: Lactate Dehydrogenase
Alb: Albumin
AST: Aspartate Aminotransferase
ALT: Alanine Aminotransferase
TBL: Total Bilirubin
Fib: Fibrinogen
ESR: Erythrocyte Sedimentation Rate
HFNC: High-Flow Nasal Cannula
PE: Plasma Exchange
